# A model for warfare in stratified small-scale societies: The effect of within-group inequality

**DOI:** 10.1371/journal.pone.0188970

**Published:** 2017-12-11

**Authors:** Sagar Pandit, Gauri Pradhan, Carel van Schaik

**Affiliations:** 1 Department of Physics, University of South Florida, Tampa, Florida, United States of America; 2 Anthropological Institute & Museum, University of Zurich, Zurich, Switzerland; Rijksuniversiteit Groningen, NETHERLANDS

## Abstract

In order to predict the features of non-raiding human warfare in small-scale, socially stratified societies, we study a coalitionary model of war that assumes that individuals participate voluntarily because their decisions serve to maximize fitness. Individual males join the coalition if war results in a net economic and thus fitness benefit. Within the model, viable offensive war ensues if the attacking coalition of males can overpower the defending coalition. We assume that the two groups will eventually fuse after a victory, with ranks arranged according to the fighting abilities of all males and that the new group will adopt the winning group’s skew in fitness payoffs. We ask whether asymmetries in skew, group size and the amount of resources controlled by a group affect the likelihood of successful war. The model shows, *other things being equal*, that (i) egalitarian groups are more likely to defeat their more despotic enemies, even when these are stronger, (ii) defection to enemy groups will be rare, unless the attacked group is far more despotic than the attacking one, and (iii) genocidal war is likely under a variety of conditions, in particular when the group under attack is more egalitarian. This simple optimality model accords with several empirically observed correlations in human warfare. Its success underlines the important role of egalitarianism in warfare.

## Introduction

Warfare can be seen as coordinated coalitionary aggression between social units. Whereas it is common among eusocial animals [1], its taxonomic distribution among other animals, including nonhuman primates, is patchy [2,3]. However, among humans, it is a cultural universal [4], and therefore probably has been present since our species arose [5] and most likely even well before [6].

Human warfare is quite variable in its expression [5,7,8]. When our ancestors were all still nomadic foragers and thus lived in fission-fusion societies without fixed settlements, warfare was predominantly in the form of raids [4,6]. Raiding can be modeled using a behavioral-ecology approach [9] similar to the one used in nonhuman species at both the individual [10] and coalition levels [11,12,13], where coalitions are defined as simultaneous and coordinated attacks by two or more partners on a common target. These functional models assume that natural selection has favored the evolution of information-gathering and decision-making mechanisms by individuals that optimally serve their fitness interests [14]. This approach revealed fundamental functional similarities between the raids of human foragers and chimpanzees, and outlined the conditions likely to favor raiding [9,15] as well as more detailed predictions about who is targeted [16]. Its success supports the assumption that men pursue their individual fitness interests when raiding [17].

After the origin of sedentism and especially agriculture, people began to live in permanent villages and over time chiefdoms arose. Thus, societies became more socially stratified and the role of power and coercive ability increased [18,19]. War changed with it, and could now also frequently involve battles and campaigns, rather than mainly surprise raids. Its aim was to acquire real estate, fertile land, and food stores [5,8,20], and sometimes women or slaves. War thus became complex war [6], characterized by hierarchical command structures and attempts to occupy the land of the other group or subjugate them.

Here, we model this war in small-scale, socially stratified societies using the same coalition models as used before for raiding. We assume that individuals maximize their fitness, as is usually done for animals, rather than group fitness [21,22]. The model is not a classic evolutionary model, and thus does not present dynamics or kinematics of individual actions. Instead, we consider the average behavior of an ensemble of equivalent groups of individuals as equilibrium snapshots, by generating numerous realizations of the model situations. This type of approach is quite common in statistical physics. Thus the model presents a potentially static equilibrium situation before and after a large number of conflicts have occurred (essentially a picture after the dust settles). In the interest of simplicity we assume that all the killings in the model are intentional. This does not mean participants never die accidently, but because such stochastic events are likely to happen at all the parameter values, we assume, they should not affect the comparisons in the model. Our primary aim is to understand the possible effects of within-group inequality in fighting ability or wealth on the outcome of war, including the likelihood that war turns genocidal, and to examine the role of defection.

The reason for limiting our scope to small-scale societies is that the simplifying assumptions made by behavioral-ecology models are far more likely to hold there than for modern wars between states [5]. These assumptions are that each individual makes independent decisions (even if participation could be socially influenced [23], but see [17,6], the intensity of an individual’s participation would still partly reflect his independent decision); that groups do not exact tribute and thus do not impose coercive taxes on neighboring societies; that there are no mercenaries or professional armies and thus no extrinsic influences of wealth on individual or coalitional strength; and that each man uses simple weapons, and there is thus no special class of trained warriors with special weapons.

Before moving onto the details of our model we would like to point to some other mathematical models that studied the effect of a collective action problem within groups of rational individuals on intergroup conflict [24,25]. There are also agent-based computational models that focus on within-group skew on aggression [26,27,28]. Although these models are interesting in their own right, they do not investigate the dependence of intergroup conflict on within-group skew, which is the key feature of our model.

## The basic assumptions of the model

We consider war as a coalition of men fighting another coalition of men, in contrast to most social-science models, which sidestepped the within-group dynamics by using groups as the unit of analysis (e.g. [29,30]. The focus on men is justified by the fact that historically men were the major participants [31], although this does not imply that women did not play a role in inciting men to go to war or refrain from it [17]. The model assumes that men compete for access to resources that limit fitness, and that a man’s fighting ability affects this access. Here we assume that fighting ability, power, and dominance rank will be highly inter-correlated and use these terms interchangeably.

**Fig 1:**
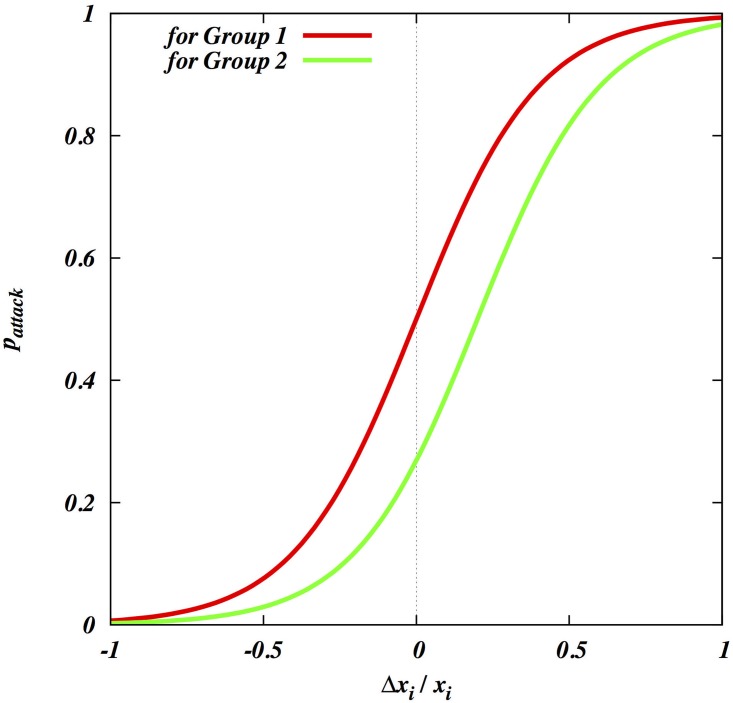
The probability that an individual male joins the attacking coalition of the willing as a function of his expected proportional change in payoff. The upper line (red) shows the function for members of group 1 (without loss of generality assumed to be the attacking group); the lower line (green) shows it for defectors from group 2. The probability of joining the attack by a defector from group 2 (without loss of generality assumed to be the defending group) is considered to be little lower than that for the members from group 1. However, because most defectors are lower to mid rankers from group 2, the exact reduction in probability does not produce a significant change to the net fighting ability of the attacking coalition.

We will assume that war is viable for the attackers only if two conditions, profitability and feasibility, are met simultaneously for each participating individual [11]. War must be profitable: it must bring a net benefit to the individual participants in the attacking coalition, i.e. an improvement in post-war (annualized) fitness relative to pre-war fitness, based on the long-term expectation after numerous realizations of the model. A male for whom war is not profitable will decide not to participate. Thus we postulate that the probability of participation of an individual is a sigmoid function of the expected net benefit (Fig 1). This means we are interested in the changes in payoff of an individual after war relative to that before war. Thus, rather than on absolute payoff, we are focused on the distribution of relative payoffs and its dependence on model parameters like within-group skew.

**Fig 2:**
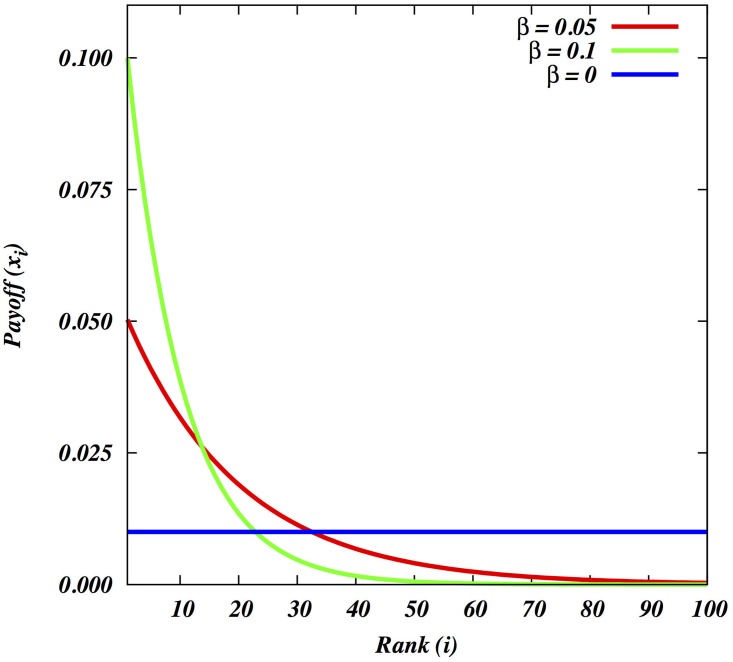
Illustrating the effect of reproductive skew, *β*, for N = 100. *β* = 0 represents perfect scramble where payoff is distributed equally among the group members. For primate and human societies typically a *β* of 0.1 or above represents a fairly despotic group, since almost 50% group members have close to zero payoff.

The magnitude of the economic benefit due to successful attack or defense among individual men varies in relation to their power in society, and in a natural-fertility population will be translated into variable fitness benefits [32,33]. Because in small-scale societies there is a variable incidence of polygyny [34] and the link between power and fitness is especially strong in polygynous societies, we assume a positive correlation between an individual man’s power or fighting ability and reproductive success [35, 36]. Accordingly, we use a fitness-skew measure based on priority of access [37], where a male’s power, or dominance rank, directly corresponds to access to reproduction, resulting in a relationship between power and instantaneous fitness that is concave (negative exponential) or at best linear, but never convex. This fitness-skew measure, *β*, is a measure of within-group inequality [11,38]. Please note that it is defined similarly as the *λ* of reference [38]. It varies between 0 and 1, with low values signifying egalitarianism and high ones despotism. In human societies of the size considered here, realistically, *β* can vary between 0.05 and 0.2 (Fig 2).

War must also be feasible: the attacking coalition’s total fighting ability must exceed that of the defensive coalition. The latter is composed of those for whom defense would be profitable, i.e. where the status quo brings a greater fitness benefits than the post-war expectation. The essential criterion for the viability of war is therefore that the coalition of the willing in the attacking group (which may potentially include some members of the defending group who switched; see below) will defeat the coalition of the willing in the defending group (which again may sometimes include a few members of group 1). Since the payoff of an individual depends on his rank and rank is determined by his fighting ability, we assume that the payoff follows the same monotonically decreasing functional form (with respect to the rank index) as fighting ability. However, it is characterized by a separate parameter, which allows us to relax this assumption if needed.

The *viability index* is defined as the difference in summed fighting ability of the men willing to join the coalition between group 1 and group 2, where the willingness to join is a probability function of expected fitness benefit. In the following we use the viability index as our estimate of the probability of a successful war.

We will assume that the eventual outcome of a successful aggressive war is a merged group containing the surviving members of both original groups, in which each man is ranked according to his intrinsic fighting ability. The merged group arises either after the majority of men in the defeated group or only some of their elite are killed [5]. We call the first kind genocidal “all-out war” and the second kind regular “all-out war” war (the term all-out being added to distinguish it from raiding warfare [9]), and include the number of enemies killed as an important free parameter into the model. Ethnography shows there are many cases of small-scale societies where a defeated society was merged into the victorious society, even if the process took some time [22,39].

Finally, we must insert a few remarks to avoid confusion. First, we do not incorporate genetic relatedness into the models (but see Discussion). In many small-scale societies, because at least some degree of exogamy was probably common, allied men need not be closely related [40], especially in the larger, somewhat stratified ones modeled here. Indeed, nearby societies were only weakly genetically differentiated [41,17]. Moreover, our results generally will not change qualitatively if kinship among the men were included.

Second, this approach does not mean that conflicts should never occur because the outcome is already known in advance. Real-world individuals have imperfect information [42] and do not engage in explicit cost-benefit analysis. Instead, they use cues honed by natural selection and cultural evolution so that the average outcome over many cases turns out to be close to optimal. As a result, the model should summarize the average or net outcome of an infinite number of cases. So in practice, unsuccessful wars (i.e. wars that never would be fought by individual rational agents) should also be expected, but because such wars maintain the status quo, we do not consider them.

## The formal model

Based on the stated assumptions we write a formal model that focuses on conflicts between two distinct groups: group 1 (without loss of generality always assumed to be the attacker) and group 2 (always assumed to be the defender), with *N*_1_ and *N*_2_ individuals, respectively. We assume that every individual in each group has freedom of choice when it comes to joining the war.

The groups compete over shareable and divisible resources, such as access to females, land or food stores. The total amount of this resource for group 1 is *X*_1_ (jointly held by *N*_1_ members) and for group 2 is *X*_2_ (jointly held by *N*_2_ members). Each individual male has a distinct dominance rank that is determined exclusively through his fighting ability within his own group (which is a combination of intrinsic strength, access to technology, skill with weapons and social abilities). For some individuals in some human societies, dominance rank may be determined by factors other than fighting ability (such as prestige: [33]). Such individuals may not be good fighters but as long as their number does not depend on the parameter *β*, we can safely average out their effect. The dominance rank determines their share of the resources in their respective groups. Thus, the *i*^*th*^ ranked individual in group 1, has $X_{1}^{i}$ resources available, such that the total resources of the group 1 are

$$X_{1}=\sum_{i=1}^{N_{1}}x_{1}^{i}$$

Similarly, for group 2,

$$X_{2}=\sum_{i=1}^{N_{2}}x_{2}^{i}$$

The model assumes that war is profitable only if it brings a net benefit to the individual participants. Thus we need to have a way to determine the payoff that each male is entitled to, based on his dominance rank before the war [11]. We assume that the male’s payoff is not affected by the males ranking below him and has a very weak dependence on the total number of males. This gives rise to a negative exponential payoff curve with rank (as is confirmed in studies of non-human primates [37,43,44]). We refer to this measure *β* as within-group inequality, which varies from 0 (complete egalitarianism or pure scramble) to 1 (absolute despotism or contest, i.e., absolute monopolization for the top-ranking male). *β* is defined as the proportion of resources left over by the next-higher-ranked individual that is usurped by the focal individual (see [11,45].

We thus arrive at the relationship between the dominance rank and resources acquired (payoffs) by each male for group 1 and 2, which is a geometric series and is given by

$$x_{1}^{i}=\frac{X_{1}\beta_{1}{\left(1-\beta_{1}\right)}^{\left(i-1\right)}}{1-{\left(1-\beta_{1}\right)}^{N_{1}}}$$

and

$$x_{2}^{i}=\frac{X_{2}\beta_{2}{\left(1-\beta_{2}\right)}^{\left(i-1\right)}}{1-{\left(1-\beta_{2}\right)}^{N_{2}}},$$

where the parameters *β*_1_ and *β*_2_ are the degree of economic, and therefore reproductive, skew for group 1 and group 2, respectively. This measure varies among human societies, from low among mobile foragers to very high (despotism) in early states [46,47,48]. Nonetheless, values of *β* exceeding 0.25 should be quite rare in small-scale human societies.

Successful attack is feasible if the attacking group’s total fighting ability exceeds that of the defending group. In order to evaluate this condition, we need a way to add up the fighting abilities of the individual players [11,49]. Here we assume that the group’s fighting ability or strength, *S*_*j*_, is the simple sum of the individual male’s fighting abilities ($s_{j}^{i};j=1,2$ in our case) of those individuals that decided to participate, as follows

$$S_{j}=\sum_{i=1}^{N_{j}}s_{j}^{i}$$

Although this assumption ignores the various ways in which the combined strength could be hampered or synergized [13], it was found empirically to work well for small coalitions of nonhuman primate males [50]. Moreover, minor variations in this assumption are not likely to affect the results much. We also assume that the men participate without coercion, although in the presence of coercion we could simply assume that a conscripted man’s willingness to actually fight depends on his prospective net fitness benefit.

The relationship between a male’s rank *i* in a group *j* and his fighting ability $s_{j}^{i}$ follows priority of access as well, and is represented as

$$s_{j}^{i}=\frac{S_{j}\sigma_{j}{\left(1-\sigma_{j}\right)}^{\left(i-1\right)}}{1-{\left(1-\sigma_{j}\right)}^{N_{j}}}$$

The parameter *σ*, similar to *β*, describes the relationship between an individual’s fighting ability and his rank. An individual’s payoff and his fighting ability are expected to show similar relationships with dominance rank, especially when *β* is modest [49]. Therefore, we assume *β* and *σ* to co-vary. If the skew is very steep (very high *β*), the difference in payoff may exceed the difference in fighting ability. But since we are dealing with societies that have low values of *β*, we can equate the two in order to simplify the calculations (although this is not a requirement of the model).

The model thus has ten external parameters viz., *N*_1_, *N*_2_, *X*_1_, *X*_2_, *S*_1_, *S*_2_, *β*_1_, *β*_2_, *σ*_1_ and *σ*_2_.

In war, group 1 attacks group 2, and if successful, subdues group 2. To keep the mathematics as simple as possible, we assume that in the process, group 1 targets the top *N*_*k*_ (such that 0 ≤ *N*_*k*_ ≤ *N*_2_) members of group 2 for elimination, so the new merged group has *N*_1_ + *N*_2_ − *N*_*k*_ individuals, with total resource base of *X*_1_ + *X*_2_ distributed according to *β*_1_. Thus, we assume no accidental killing and no casualties on the attacking side. However, as discussed below, adding costs will not fundamentally change the findings.

Every male in either group joins the war provided both the profitability and feasibility conditions are satisfied. Note that the profitability condition must be satisfied at the individual level, but the feasibility condition at the level of the coalition. Every participant from both the attacking side (Group 1) and the defending side (Group 2) thus computes his current as well as new payoff in order to decide whether to join the war and whether it is on the defending or the attacking side. The success of the war for either group is dependent on the ability to gather enough fighting ability.

We assume that group 1 attacks group 2 and kills *N*_*k*_ (where *N*_*k*_ ≤ *N*_2_) individuals from group 2 starting from the top ranker (See [4]). The remaining members of group 2 join group 1 according to their fighting ability where this newly formed group has its own new *β*, which for simplicity of presentation we assume to be the same as the original *β*_1_ of the attacking group. For the new group, the size of the group and the total resources are as follows:

$$N=N_{1}+N_{2}-N_{k}$$

$$X=X_{1}+X_{2}$$

We first calculate the ranks of individuals in both the groups based on their fighting abilities.

$$s_{1}^{i}=s_{2}^{j}$$

$$\frac{S_{1}\sigma_{1}{\left(1-\sigma_{1}\right)}^{\left(i-1\right)}}{1-{\left(1-\sigma_{1}\right)}^{N_{1}}}=\frac{S_{2}\sigma_{2}{\left(1-\sigma_{2}\right)}^{\left(j-1\right)}}{1-{\left(1-\sigma_{2}\right)}^{N_{2}}}$$

Let $\frac{S_{1}}{S_{2}}=M$ and $\frac{\sigma_{1}}{\sigma_{2}}=\mu$, which gives the rank in group 2 for the *i*^*th*^ ranking individual in group 1 as

$$j=(i-1)\frac{\text{ln}(1-\sigma_{1})}{\text{ln}(1-\sigma_{2})}+\frac{\text{ln}(M\mu B)}{\text{ln}(1-\sigma_{2})}+1$$

whereas the rank for the *j*^*th*^ ranking individual in group 2 if he were placed in group 1 based on his fighting ability is

$$i=(j-1)\frac{\text{ln}(1-\sigma_{2})}{\text{ln}(1-\sigma_{1})}-\frac{\text{ln}(M\mu B)}{\text{ln}(1-\sigma_{1})}+1,$$

where

$$B=\frac{1-{\left(1-\sigma_{1}\right)}^{N_{1}}}{1-{\left(1-\sigma_{2}\right)}^{N_{2}}}$$

We can now compute the new ranks of all the individuals after the final group merger. Now, the (*N*_*k*_ + 1)^*th*^ individual is the first from group 2 to join the new group. Using the calculation above we can write the new rank of this (*N*_*k*_ + 1)^*th*^ individual from group 2 in the merged group by substituting *j* = *N*_*k*_ + 1 as

$$N_{k}{}^{\left(1\right)}=N_{k}\frac{\text{ln}(1-\sigma_{2})}{\text{ln}(1-\sigma_{1})}-\frac{\text{ln}(M\mu B)}{\text{ln}(1-\sigma 1)}+1$$

The new rank of previously *i*^*th*^ ranking individual becomes *i*^*new*^ = *i* if $i\leq N_{k}^{\left(1\right)}$

If $i>N_{k}^{\left(1\right)}$ then $j=(i-1)\frac{\text{ln}(1-\sigma_{1})}{\text{ln}(1-\sigma_{2})}+\frac{\text{ln}(M\mu B)}{\text{ln}(1-\sigma_{2})}-N_{k}$ individuals from group 2 are going to be inserted above him. Here we assume that the individual in group 2 with same fighting ability as an individual from group 1 is inserted below. So we get

$$i_{0}^{new}=(i-1)+(i-1)\frac{\text{ln}(1-\sigma_{1})}{\text{ln}(1-\sigma_{2})}+\frac{\text{ln}(M\mu B)}{\text{ln}(1-\sigma_{2})}-N_{k}+1$$

Therefore we can write that for the individuals in group 1

$$i^{new}=\{\begin{array}{c} \begin{array}{ccc} i & if & i\leq N_{k}^{\left(1\right)} \end{array}\\ \begin{array}{ccc} i_{0}^{new} & if & i>N_{k}^{\left(1\right)} \end{array} \end{array}$$

For group 2 individuals, if the rank *j* ≤ *N*_*k*_ then, *i*^*new*^ = ∞ (they are killed), whereas if *j* > *N*_*k*_ then

$$i_{00}^{new}=(j-1)\frac{\text{ln}(1-\sigma_{2})}{\text{ln}(1-\sigma_{1})}-\frac{\text{ln}(M\mu B)}{\text{ln}(1-\sigma_{1})}+2+j-N_{k}$$

As a result, for the individuals from group 2 will have new rank as

$$i^{new}=\{\begin{array}{c} \begin{array}{ccc} i & if & i\leq N_{k}^{\left(1\right)} \end{array}\\ \begin{array}{ccc} i_{0}^{new} & if & i>N_{k}^{\left(1\right)} \end{array} \end{array}$$

Thus the new payoff received by an individual in group 1 is given by

$$x_{i}\prime =\{\begin{array}{ccc} \frac{(X_{1}+X_{2})\beta_{1}{\left(1-\beta_{1}\right)}^{i-1}}{1-{\left(1-\beta_{1}\right)}^{N_{1}+N_{2}-N_{k}}} & if & i\leq N_{k}^{\left(1\right)}\\ \frac{(X_{1}+X_{2})\beta_{1}{\left(1-\beta_{1}\right)}^{i_{0}^{new}-1}}{1-{\left(1-\beta_{1}\right)}^{N_{1}+N_{2}-N_{k}}} & if & i>N_{k}^{\left(1\right)} \end{array}$$

and the new payoff received by the individual in group 2 is

$$x_{i}\prime =\{\begin{array}{ccc} 0 & if & i\leq N_{k}\\ \frac{(X_{1}+X_{2})\beta_{1}{\left(1-\beta_{1}\right)}^{i_{00}^{new}-1}}{1-{\left(1-\beta_{1}\right)}^{N_{1}+N_{2}-N_{k}}} & if & i>N_{k} \end{array}$$

The probability of joining the attack is postulated as a sigmoid function

$$p(k,\theta ,\Delta x_{i})=\frac{1}{1+e^{-k(\Delta x_{i}-\theta )}}$$

(See Fig 1), where $\Delta x_{i}=\frac{x_{i}^{\prime }-x_{i}}{x_{i}}$, *k* is the parameter that estimates the risk-taking ability of the group (the higher *k*, the more likely the individual risks joining), and *θ* is the percentage offset in payoff used for individuals that decide to join the opponent’s coalition as a defector (we will refer to them as *switchers* to avoid confusion). Similarly the probability of joining the defensive coalition is postulated as 1 − *p*(*k*, *θ*, Δ*x*_*i*_). The actual values of *k* and *θ* do not play a significant role in qualitative outcome of the models.

The cost of war may be introduced through the parameter *θ* (as it is set higher for switchers). Again, its value does not really alter the qualitative outcome because usually the switchers are low to mid rankers in either group, whose contributions do not significantly alter the coalitions’ fighting abilities. It is extremely difficult to assess components of costs in human society, so instead of introducing an arbitrary cost we decided to investigate the limiting case of the zero-cost model. As long as costs are weakly dependent on model parameters like *β*, the model will overestimate the likelihood of warfare and can be used as an upper bound. Thus, if costs become higher for everyone, war will be found at more extreme parameter values than reported below. If, however, costs increase with reduced fighting ability, as seems reasonable, then the model’s conclusion will become even stronger. In both these situations, we may get qualitative results similar to those found in the upper-bound case considered here.

Based on the probability of joining the fighting coalitions we write the fighting ability of the attacking coalition as

$$FA_{a}=\sum_{i=1}^{N_{1}}s_{1}^{i}p(k,0.0,\Delta x_{i})+\sum_{j=1}^{N_{2}}s_{2}^{j}p(k,0.6,\Delta x_{j})$$ (1)

and that of the defensive coalition as

$$FA_{d}=\sum_{i=1}^{N_{1}}s_{1}^{i}(1-p(k,-0.6,\Delta x_{i}))+\sum_{j=1}^{N_{2}}s_{2}^{j}(1-p(k,0.0,\Delta x_{j})).$$ (2)

The success of war depends on the value of the *feasibility index*, Δ − *FA*_*a*_ − *FA*_*d*_. If Δ is positive, then war is more feasible with higher values of Δ. We note that the second term in Eq (1) and the first in Eq (2) are due to individuals defecting and joining the fights on the opponents’ side. In the following analysis, whenever we describe situations without defection we will be setting these terms to zero. The qualitative trend for Δ does not depend on the value *θ* = 0.6, although for clarity of presentation this particular value is used when plotting figures.

## Results

**Fig 3:**
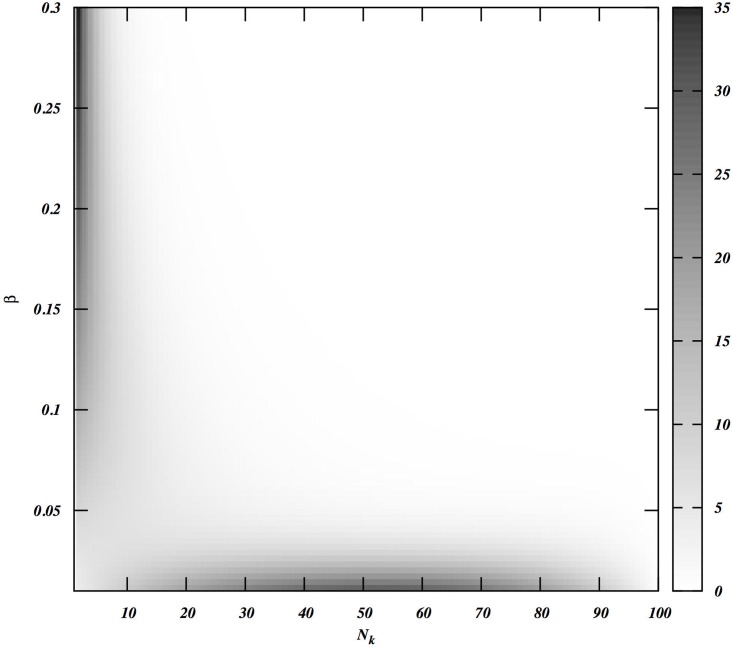
The viability index (Δ) of group 1’s attacks as a function of the skew (*β*; equal in both groups) and the number of enemy men eliminated (N_k_), when groups are equal in all respects.

We first examine the role of within-group skew on warfare. We will assume that both the groups are identical in all respects. Fig 3 describes this situation, showing the density plot of Δ as a function of *N*_*k*_ and *β*_1_. We note that the feasibility index for war is very low under these conditions. The only regions where the feasibility index is high enough to present a war option are (a) high *β*_1_ and low *N*_*k*_ region where the war eliminates only the top fighter(s) in group 2 when both groups are highly skewed; or (b) low *β*_1_ and high *N*_*k*_ region where the war eliminates a large number of men (always focusing on higher-ranking men where possible) when both groups are highly egalitarian. In the absence of defection, the essential picture does not change but war becomes even more unlikely.

**Fig 4:**
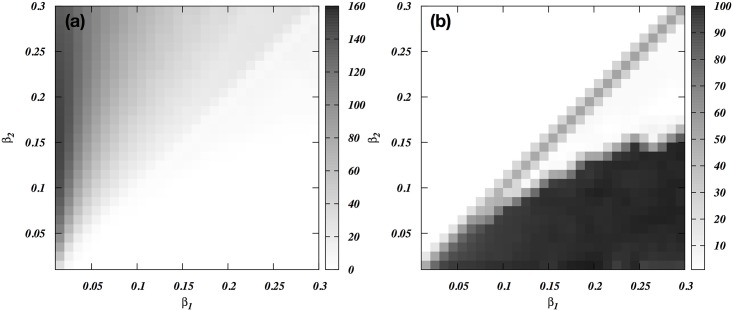
(A) The viability index (Δ) of group 1’s attacks as a function of the skew (*β*) in the two groups. (B) The number of men in group 2 eliminated (N_k_) that maximizes group 1’s viability index.

**Fig 5:**
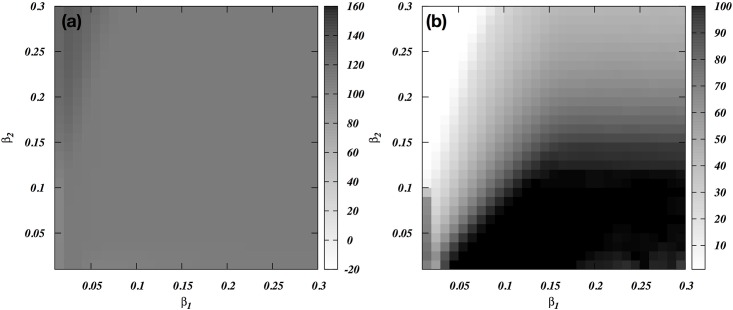
(A) Group 1’s viability index (Δ) for the situation where it has twice the maximum total fighting ability of group 2, but only half the population (S_1_ = 200; N_1_ = 100; S_2_ = 100; N_2_ = 200). (B) The value of N_k_ at which the viability index is maximized).

When there is a major discrepancy between the skew in the two groups, but the groups are otherwise equal, the following results arise. First, low-skew groups can easily defeat otherwise similar high-skew groups (Fig 4a), and in the process only eliminate the elites from the defending group, or even the leader only (Fig 4b). Second, it is unlikely for high-skew groups to defeat low-skew groups, but if they can they will eliminate many members of group 2. However, if group 1 has superior fighting ability, e.g. because they have superior weapons, it can defeat larger groups with less effective weapons. Fig 5a shows that, not surprisingly, they can easily defeat group 2 at each combination of skews. However, as shown in Fig 5b, they will nonetheless end up eliminating most men of group 2 if the defending group is highly egalitarian. Thus, when small groups with superior weaponry attack larger groups with more primitive arms, they can easily win, but to maximize profit they may decide to completely eliminate males from the other group.

### What is the role of switchers?

**Fig 6:**
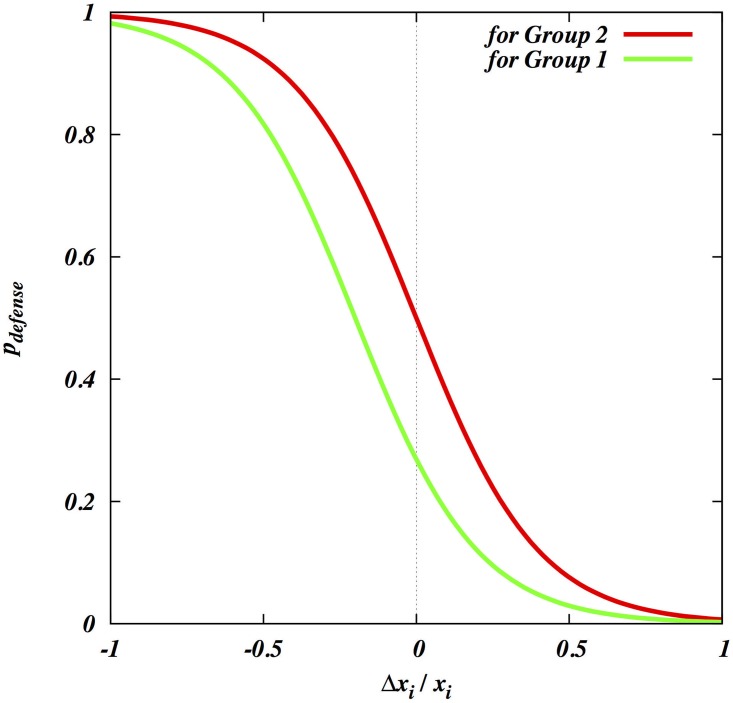
The probability of joining the defensive effort by group 2’s coalition of the willing as a function of an individual’s expected proportional change in fitness. The upper line (red) shows the function for members of group 2; the lower line (green) shows it for switchers from group 1.

**Fig 7:**
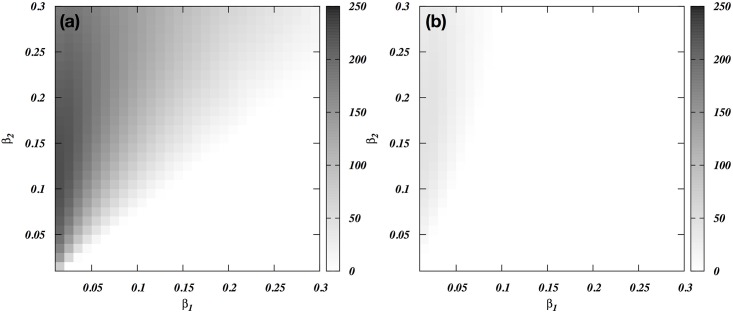
(A) The viability index (Δ) of group 1’s attacks when group 2 is twice as strong as group 1, as a function of within-group skew (*β*) in each group. Switching is allowed. (B) The viability index of group 1’s attacks when group 2 is twice as strong as group 1, as a function of within-group skew in each group. Here, however, no switching is allowed.

The model allows individuals from group 2 to defect and join group 1, if this is profitable for them. We have added a clear threshold for doing so, assuming that switching is costly in various ways (see Figs 1 and 6). By repeating all analyses with and without switching, we found that it has a modest role in most cases. The exception is when the attacking group 1 has a lower total fighting ability but defending group 2 has higher skew. Then, the sub-elite (mid rankers) of group 2 is tempted to switch. To illustrate this, we set group 2’s maximum total fighting ability to twice that of group 1. In Fig 7a, where we allow switching, group 1 can defeat group 2 if it has very low skew whereas the targeted group has very high skew. As expected, Fig 7b shows that this effect virtually disappears when we do not allow switching.

## Discussion

We modeled features of warfare in sedentary, stratified, small-scale human societies with modest asymmetries in technology or size. The model assumes that each individual independently decides on participation based on economic benefits, which translate into fitness benefits. Making these simplifying optimality assumptions, the model made some predictions about the conditions leading to regular or genocidal war among such groups.

We recognize that human war is complicated, and that any model inevitably omits potentially relevant factors. For instance, our model does not make predictions about how protracted or how difficult wars will be, except that if the difference in total fighting ability is smaller, the wars are more likely to be protracted and expensive for the attackers. The most important simplifying assumption is that engaging in war itself has no cost because it is extremely difficult to estimate them. We can, however, explore various scenarios. On one hand, if we assume realistically that an individual’s cost decreases with increasing fighting ability, our conclusions will hardly be affected. On the other hand, introducing a flat cost will make all-out wars less likely. Thus, we can consider the cost-free war situation as the upper bound on the probability estimation of war. As seen from Fig 4, all-out wars are very rare and thus when costs are introduced, we expect the all-out wars will be even less likely to occur. Although this might mean that in practice no wars will ever happen at many parameter values, the true test of the model is to assess the extent to which it captures the essential features of all-out warfare in small-scale societies, by comparing its results with actually observed patterns.

### Skew

Low-skew, i.e. egalitarian, societies can defeat high-skew, i.e. despotic, societies when all else is equal (Fig 5), and even when the defending group is bigger or stronger (Fig 7). This result arises because elites in high-skew societies have trouble recruiting males into the coalition of the willing because the lower-ranking males are less likely to benefit from a successful war. This conclusion obviously depends on the assumption that elites cannot coerce individuals to participating in wars. Another critical assumption was that skew in fighting abilities equates skew in wealth and reproductive fitness. That societies vary greatly in skew in intrinsic fighting abilities is unlikely. However, training with specialized weapons and hoarding them may increase skew. In addition, leverage and payments may make some men far more powerful than based on their intrinsic fighting ability, and with increasing complexity of societies these factors become increasingly important. This assumption also allows us to assume that over time the combined groups will acquire the skew in power of the winning group.

Human societies seem to exhibit scale-independent properties [26], which allows us to speculate about extending the model results to more complex societies. In such societies, leaders can politically decide to change a society’s skew in wealth. This implies that equating the two may not always be justified, but where politics raises the stakes of being higher-ranked in society, we expect men to rapidly adjust, by training to use weapons, by buying expensive weapons, or by hiring people to help in fighting. Thus, the assumption of rapid adjustment of skew in fighting ability to that in wealth appears to be warranted. Overall, skew increased as societies evolved from the macro-bands or communities of mobile foragers to sedentary societies of complex foragers and the tribal societies of extensive farmers, and subsequently to chiefdoms, the kingdoms of early states, and finally the highly despotic empires that followed [51,34]. Despite their higher skew, these more complex societies have historically overwhelmed their less complex neighbors, almost certainly because they also showed strong increases in the size of social units and their technology, and thus total fighting ability.

War-prone societies tend to be high-skew. One clear prediction for real-world wars therefore is that societies setting out to conquer neighboring territory should have a clear superiority in terms of numbers or weapons, especially if the groups they target generally have lower-skew. This prediction is consistent with observation [52].

### Skew and genocide

Skew also plays a major role in that it affects which members of the enemy group are eliminated during successful attacks. Indeed, perhaps the most surprising model result is that in many cases, victory over the enemy group involves eliminating many of the men, even when the attacking group can easily defeat the defending group. The elimination can be in the form of killing these men, enslaving them and not allowing them to reproduce, or forcing their emigration. Perhaps counter-intuitively, this becomes more likely with increasing size of the defending group.

How realistic is this prediction from the model? Where men are essential for the production of *X* (the group’s food base or tradable materials), as in forms of agriculture that essentially require men’s labor inputs, the conclusions of the model may not apply, because eliminating many men may actually reduce *X*_2_. On the other hand, genocidal wars are to be expected among small-scale societies in regions with rapidly expanding populations. The model’s conclusion therefore applies whenever victorious group 1 has a surplus of young men, who would otherwise not be able to inherit land or other property, and would be especially interested in acquiring land, if women or slaves can produce the food, or in the case of sedentary foragers. This finding is actually quite consistent with the emerging consensus in the literature that warfare among small-scale horticultural societies was common and occasionally very violent. Archeology increasingly shows that the wars among rapidly expanding or migrating Neolithic farmers and between them and foragers at least occasionally involved the mass slaughter of both combatants and non-combatants [53,54]. Moreover, ethnography shows that many non-foraging, small-scale societies are known to engage in violent warfare [4, 5,31].

Once more, extending model predictions to more complex societies provides a fit with historically known contexts of genocide. Genocide is expected where large, rapidly expanding and technologically advanced societies begin to colonize regions with small-scale groups with more primitive weaponry, especially where the latter had little wealth and could therefore not be exploited in other ways. This description fits with the western colonization of northern America or Australia [46].

In regions with reduced egalitarianism, the attacking group can generally win by eliminating the elite only. In these situations, most men and their families will be spared. Thus, we speculate that the extreme fear of civilians for war today may no longer reflect current risk of being subject to genocide, but was highly adaptive during most of history. When people lived in societies with limited stratification, as in communities and tribes, the model predicts that war may often have resulted in genocide.

### The role of defection or switching

Switching (defection or treason, where men transfer into enemy groups to improve their position) may be difficult because it involves the double cost of leaving behind family and friends, and thus important economic ties, and exposing the switchers to possible retribution from the group’s leaders. This is why the model imposes an extra threshold on switching, even if in the model’s internal logic it would pay to do so. In the model, we found that switching from defender to attacker is especially likely when the defending group has a higher skew; the switchers should be the ones just below the elite. In low-skew societies, switching will very rarely bring any benefits.

The paucity of references to switching in the major reviews [4,5,31] suggests that the ethnographic literature is largely silent on these issues. The most plausible interpretation is that it was quite rare in small-scale societies and instead a phenomenon that first emerged with the origin of states, in which lineage affiliation was far less critical to fitness. This observation is consistent with the model’s findings, since small-scale societies are generally low-skew.

### Implications for the evolution of war

Even though our model exclusively considers economic costs and benefits and thus ignores cultural, religious, and ideological factors, it produced a good fit with the broad patterns of ethnography and archeology, suggesting that it may be an adequate description of real-world events. And although the model is explicitly written for small-scale stratified societies and in spite of many seemingly fundamental differences between small-scale and large-scale societies [34], the correspondence between the model’s predictions and empirical patterns in wars between modern states is surprisingly good. This correspondence suggests that the emotionally based, but originally adaptive decision-making rules used by people in states have remained very similar to those governing pre-state warfare [55].

The mode of natural selection that produced warfare in our evolutionary history is currently the subject of intensive debate. In our model, the group-level action of war is exclusively the product of individuals making decisions that maximize their own fitness without representing the group’s fate as such. This approach thus complements models that invoke group-level selection phenomena, in which individuals inside groups behave altruistically (have a reduced fitness relative to others in their group) because they create public benefits, such as classic group selection [21,56] or cultural group selection [57]. In light of our model, within-group egalitarianism may be favored by individual selection if war is common. If low within-group skew is based on an active choice by the elite rather than ecologically imposed, this implies that the elite reduced it in order to change the payoff of defense for the rest of the group and so recruit them to participate in a defensive war. The model can thus be seen as an alternative to the group selection models because this altruism by the elite should be flexible, leading them to reduce their takings only when the group is under external threat. The critical difference between the two approaches, whether dominants gain or lose fitness from participating in winning wars, may be virtually impossible to test. However, the present model makes testable predictions about the role of within-group skew, the conditions favoring genocide and the occurrence of switching, which are not generated by other models.

An alternative model [58] relied on kin selection to explain costly participation in war, especially the evolution of bravery and belligerence, which in the absence of war would be costly traits. Our model does not explicitly include nepotistic effects. However, our conclusions will hold for any group, whether containing kin or non-kin, because incorporating kin selection will automatically reduce *β*, resulting in an increase in the likelihood of a successful war. Because this result was reached without assuming variation in bravery or belligerence, it depends entirely on variation in fighting ability and payoffs.

The model by Gavrilets & Fortunato [59] also focuses on individual fitness, but differs by being an evolutionary model and by assuming that war creates a collective good (the expanded territory) shared in part by free-riders, who refrained from participating in the war. The fighters thus do better than before, but gain less than the free riders. In our model, free riding is avoided because males who would benefit from war all participate. If we modify the basic assumptions and allow some males not to be fighters, a similar free-rider problem might also arise in our model. However, because we assume that groups merge, these males would not benefit very much, if at all, from the outcome of war. Nonetheless, both Gavrilets & Fortunate [59] and our model suggest that individual-selection models of warfare can work, in line with recent similar suggestions [60].

It is well known that despotic societies are more belligerent [61]. One explanation proposed for this phenomenon is that war and higher within-group inequality go together because groups require a despotic social organization in order to be effective at all-out warfare [61,62,63]. Under the model, however, egalitarian societies are more successful in warfare than despotic groups of comparable, or even somewhat larger, size. Thus, societies in which command structures do not automatically lead to major skew will generally do better in competition. If societies became despotic for intrinsic reasons (see [64]), then despotic societies can afford to be more belligerent when they are surrounded by other despotic societies.

Finally, we hypothesize that the model offers a possible explanation for the gradual reduction of inequality recorded in states over time: human states started out highly despotic and have gradually become less so [5,65]. There is no widely accepted explanation for this phenomenon. However, based on the model we can speculate that one benefit of becoming more egalitarian is that a society becomes less prone to be attacked by neighbors and more likely to win wars. Since this contributes to a society’s survival, one would expect it to be a viable strategic shift in a process of competition among states.

## Conclusion

This model examined warfare in small socially stratified (tribal, chiefdom-like) societies entirely from an individual-selection perspective. Its results suggest that within-group inequality has a major, frequently overlooked effect on the incidence of warfare. It shows that egalitarian groups are more likely to defeat more despotic (at times even stronger) enemies, that switching to enemy groups will be rare, unless the attacked group is far more despotic than the attacking one, and that genocidal war is especially likely when the attacked group is more egalitarian. These predictions are supported empirically, but also fit well with observed patterns in modern warfare. This correspondence suggests that the rules originally favored by natural selection for the decision to go to war in small-scale societies retain much of their predictive power when it comes to warfare among more large-scale societies. Modern-day humans thus may still use the logic of within-group inequality when it comes to war, despite dramatically different economic conditions.

## References

[1] Wilson EO. The insect societies. Harvard: Harward University Press; 1971.

[2] Kitchen DM, Beehner JC. Factors affecting individual participation in group-level aggression among non-human primates. Behav. 2007; 144: 1551–1581.

[3] Willems E, Arseneau TJM, Schleuning X, van Schaik C. Communal range defence in primates as a public goods dilemma. Phil Trans Roy Soc B. 2015; 370: 20150003. 26503678 10.1098/rstb.2015.0003 PMC4633841

[4] Gat A. Proving communal warfare among hunter-gatherers: the quasi-rousseauan error. Evol Anthro. 2015; 24: 11–126. 10.1002/evan.21446 26081116

[5] Keeley LH. War before Civilization. New York: Oxford University Press; 1996.

[6] Wrangham RW, Glowacki L. Intergroup aggression in chimpanzees and war in nomadic hunter-gatherers—Evaluating the chimpanzee model. Hum Nat. 2012; 23: 5–29. doi: 10.1007/s12110-012-9132-1 22388773 10.1007/s12110-012-9132-1

[7] Vasquez JA. The War Puzzle Revisited. Cambridge: Cambridge University Press; 2009.

[8] Diamond J. The World Until Yesterday. New York: Penguin Books; 2013.

[9] Pandit SA, Pradhan GR, van Schaik CP. The conditions favoring between-community raiding in chimpazees, bonobos and in human foragers. Hum Nat. 2016; 27: 141–159. doi: 10.1007/s12110-015-9252-5 26613587 10.1007/s12110-015-9252-5

[10] Davies NB, Krebs JR, and West SA. An Introduction to Behavioural Ecology. Chichester, UK: Wiley-Blackwell; 2012.

[11] Pandit SA, and van Schaik CP. A model for leveling coalitions among male primates: Towards a theory of egalitarianism. Behav Ecol Sociobiol. 2003; 55: 161–168.

[12] Connor R, Whitehead H. Alliances II. Rates of encounter during resource utilization: a general model of intrasexual alliance formation in fission-fusion societies. Anim Behav. 2005; 69: 127–132.

[13] Mesterton-Gibbons M, Gavrilets S, Gravner J, and Akcay E. Models of coalition or alliance formation. J Theor Biol. 2011; 274: 187–204. doi: 10.1016/j.jtbi.2010.12.031 21195717 10.1016/j.jtbi.2010.12.031

[14] Maynard Smith J, and Parker GA. Optimality theory in evolutionary biology. Nature. 1990; 348: 27–33.

[15] Wilson M, Boesch C, Fruth B, Furuichi T, Gilby I, Hashimoto C, et al Lethal aggression in *Pan* is better explained by adaptive stratgeies than human impacts. Nature. 2014; 513: 414–417. doi: 10.1038/nature13727 25230664 10.1038/nature13727

[16] Pradhan GR, Pandit SA, van Schaik CP. Why do chimpanzee males attack the females of neighboring communities? Am J Phys Anthro. 2014; 155: 430–435. 10.1002/ajpa.22589 25100507

[17] Glowacki L, Wrangham R. The role of rewards in motivating participation in simple warfare. Hum Nat. 2013; 24: 444–460. doi: 10.1007/s12110-013-9178-8 24008817 10.1007/s12110-013-9178-8

[18] Chagnon N. Life histories, blood revenge, and warfare in a Tribal population. Science. 1988; 9: 985–992. 10.1126/science.239.4843.985 17815700

[19] Currie TE, Greenhill SJ, Gray RD, Hasegawa T, Mace R. Rise and all of political complexity in island South-East Asia and the Pacific. Nature. 2010; 467: 801–804. doi: 10.1038/nature09461 20944739 10.1038/nature09461

[20] LeBlanc S. Constant battles: The myth of the peaceful, noble savage. New York: St. Martin’s Press; 2003.

[21] Bowles S. Group competition, reproductive leveling, and the evolution of human altruism. Science. 2006; 314: 1569–1572. doi: 10.1126/science.1134829 17158320 10.1126/science.1134829

[22] Richerson PJ, Boyd R. Not by genes alone. Chicago: University of Chicago Press; 2005.

[23] Boyd R, Gintis H, Bowles S, Richerson PJ. The evolution of altruistic punishment. Proceedings of the National Academy of Sciences, 2003; 100: 3531–3535. 10.1073/pnas.0630443100 PMC152327 12631700

[24] Takács K. Structural Embeddedness and Intergroup Conflict. The Journal of Conflict Resolution. 2001; 45:743–769.

[25] Bornstein G. Free-rider problem in intergroup conflicts over step level and continuous public goods. Journal of Personality and Social Psychology. 1992; 62:597–606.

[26] Cederman LE. Modeling the size of wars: From billiard balls to sandpiles. American Political Science Review. 2003; 97: 135–150.

[27] Cederman LE, and Gleditsch KS. Conquest and regime change: an evolutionary model of the spread of democracy and peace. International Studies Quarterly. 2004; 48: 603–629.

[28] Hemelrijk CK. Towards the integration of social dominance and spatial structure. Animal Behaviour. 2000; 59: 1035–1048. doi: 10.1006/anbe.2000.1400 10860531 10.1006/anbe.2000.1400

[29] Bueno de Mesquita B, Morrow JD, Siverson RM, and Smith A. An institutional explanation of the democratic peace. American Political Science Review. 1999; 93: 791–807.

[30] Meirowitz A, and Sartori AE. Strategic uncertainty as a cause of war. Quarterly Journal of Political Science. 2008; 3: 327–352.

[31] van der Dennen JMG. The origin of war: The evolution of a male-coalitional reproductive strategy, Vols. 1 & 2. Groningen, Netherlands: Origin Press; 1995.

[32] Sherman PW, and Reeve HK. Forward and backward: alternative approaches to studying human social evolution In: Betzig L, editor. Human Naure: A Critical Reader. New York: Oxford University Press; 1997 p 147–158.

[33] von Rueden CR, Jaeggi AV. Men’s status and reproductive success in 33 nonindustrial scoieties: Effects of subsistence, marriage systems, and reproductive strategy. Proc Natl Acad Sci, USA. 2016; 113: 10824–10829. doi: 10.1073/pnas.1606800113 27601650 10.1073/pnas.1606800113 PMC5047206

[34] Kaplan H, Hooper PL, and Gurven M. The evolutionary and ecological roots of human social organization. Phil Trans Roy Soc, Lond B. 2009; 364: 3289–3299. 19805435 10.1098/rstb.2009.0115 PMC2781874

[35] Betzig L, Means, variances, and ranges in reproductive success: comparative evidence. Evolution and Human Behavior. 2012; 33: 309–317.

[36] Betzig L. Human nature: A critical reader. Oxford University Press: New York; 1997.

[37] Altmann SA. A field study of the sociobiology of the rhesus monkey, *Macaca mulatta*. Ann NY Acad Sci. 1962; 102: 338–435. 14012344 10.1111/j.1749-6632.1962.tb13650.x

[38] Kokko H, and Lindström J. Measuring the mating skew. American Naturalist. 1997; 149: 794–799.

[39] Henrich J. The Secret of Our Success: How Culture is Driving Human Evolution, Domesticating Our Species, and Making Us Smarter. Princeton: Princeton University Press; 2016.

[40] Hill K, Walker R, Božičević M, Eder J, Headland T, Hewlett B. et al Co-residence patterns in hunter-gatherer societies show unique human structure. Science. 2011; 331: 1286–1289. doi: 10.1126/science.1199071 21393537 10.1126/science.1199071

[41] Langergraber K, Schubert G, Rowney C, Wrangham R, Zommers Z, Vigilant L. Genetic differentiation and the evolution of coperation in chimpanzees and humans. Proc Roy Soc B. 2011; 278: 2546–2552. 10.1098/rspb.2010.2592 PMC3125631 21247955

[42] Fearon JD. Rationalist explanations for war. Intern Org. 1995; 49: 379–414.

[43] Bulger JB. Dominance rank and access to estrous females in male savanna baboons. Behav. 1993; 127: 67–103.

[44] Alberts SC, Watts HE and Altmann J. Queuing and queue-jumping: Long-term patterns of reproductive skew in male savannah baboons, *Papio cynocephalus*. Anim Behav. 2003; 65: 821–840.

[45] van Schaik CP, Pandit SA, and Vogel ER. A model for within-group coalitionary aggression among males. Behav Ecol Sociobiol. 2004; 57: 101–109.

[46] Diamond J. Guns, Germs, and Steel. New York: W.W.Norton & Company; 1997.

[47] Keeley LH. Hunter-gatherer economic complexity and "population pressure": a cross-cultural analysis. J Anthropol Archaeol. 1988; 7: 373–411.

[48] Borgerhoff-Mulder M, Bowles S, Hertz T, Bell A, Beise J, Clark G. et al Intergenerational wealth transmission and the dynamics of inequality in small-scale societies. Science. 2009: 326: 682–688. doi: 10.1126/science.1178336 19900925 10.1126/science.1178336 PMC2792081

[49] van Schaik CP, Pandit SA. & Vogel ER. Toward a general model for male-male coalitions in primate groups In: Kappeler P.M. & van Schaik CP, editors. Cooperation in Primates and Humans: Mechanisms and Evolution. Springer Verlag: Heidelberg; 2005p. 151–171.

[50] Bissonnette A, de Vries H, and van Schaik CP. Coalitions in male Barbary macaques, *Macaca sylvanus*: strength, success and rules of thumb. Anim Behav. 2009; 78: 329–335.

[51] Flannery KV. Cultural evolution of civilizations, Ann Rev Ecol Syst. 1972; 3: 399–426.

[52] Lake DA. Powerful pacifists: Democratic states and war. Amer Poli Sci Rev. 1992; 86: 24–37.

[53] Meyer C, Lohr C, Gronenborn D, Alt K. The massacre mass grave of Schöneck-Kilianstädten reveals new insights into collective violence in Early Neolithic Central Europe, Proc Natl Acad Sci, USA. 2015; 112: 11217–11222. doi: 10.1073/pnas.1504365112 26283359 10.1073/pnas.1504365112 PMC4568710

[54] Meller H, Schefzik M. (ed) Krieg: Eine archäologische Spurensuche. Landesamt für Vorgeschichte, Halle (Saale); 2015.

[55] Daly M, and Wilson MI. Human evolutionary psychology and animal behaviour. Anim Behav. 1999; 57: 509–519. doi: 10.1006/anbe.1998.1027 10196040 10.1006/anbe.1998.1027

[56] Choi Jung-Kyoo and Bowles S. The coevolution of parochial altruism and war Science. 2007; 318: 636–640. doi: 10.1126/science.1144237 17962562 10.1126/science.1144237

[57] Zefferman M, Mathew S. An evolutionary theory of large-scale human warfare: Group-structured cultural selection. Evol Anthro. 2015; 24.2: 50–61. 10.1002/evan.21439 25914359

[58] Lehmann L, and Feldman MW. War and the evolution of belligerence and bravery. Proc Roy Soc, Lond B. 2008; 275: 2877–288. 10.1098/rspb.2008.0842 PMC2605837 18755675

[59] Gavrilets S, Fortunato L. A solution to the collective action problem in between-group conflict with within-group inequality, Nature Communications. 2014; 5. 10.1038/ncomms4526 PMC3974216 24667443

[60] Glowacki L, and Wrangham R. Warfare and reproductive success in a tribal population, Proc Natl Acad Sci, USA. 2015; 112: 348–353. doi: 10.1073/pnas.1412287112 25548190 10.1073/pnas.1412287112 PMC4299188

[61] Reyna SP, and Downs RE. A mode of domination approach to organized violence In: Studying War: Anthropological Perspectives. Amsterdam: Gordon & Breach; 1994; p. 29–65.

[62] McCauley C. The Anthropology of War, Haas J., ed. Cambridge: Cambridge University Press Conference overview; 1990 p. 1–25.

[63] Turchin P, and Gavrilets S. Evolution of complex hierarchical societies. Social Evolution & History. 2009; 8: 167–198.

[64] Mattison S.M., Smith E.A., Shenk M.K. and Cochrane E.A. The evolution of inequality. Evolutionary Anthropology. 2016; 25: 184–199. doi: 10.1002/evan.21491 27519458 10.1002/evan.21491

[65] Pinker S. The Better Angels of Our Nature. New York, NY: Viking; 2011.

